# Supplementary material for: A new view on the origin of zero-bias anomalies of Co atoms atop noble metal surfaces
Source: Nat Commun. 2020 Nov 30;11:6112. doi: 10.1038/s41467-020-19746-1 (PMC7705691; doi:10.1038/s41467-020-19746-1)
Supplement: Supplementary file 1 — Supplementary Information [file 41467_2020_19746_MOESM1_ESM.pdf]

Supplementary Information: A new view on the origin of zero-bias anomalies of Co atoms atop noble metal surfaces

Juba Bouaziz, Filipe S. M. Guimarães and Samir Lounis

Peter Grünberg Institut and Institute for Advanced Simulation, Forschungszentrum Jülich and JARA, 52425 Jülich, Germany

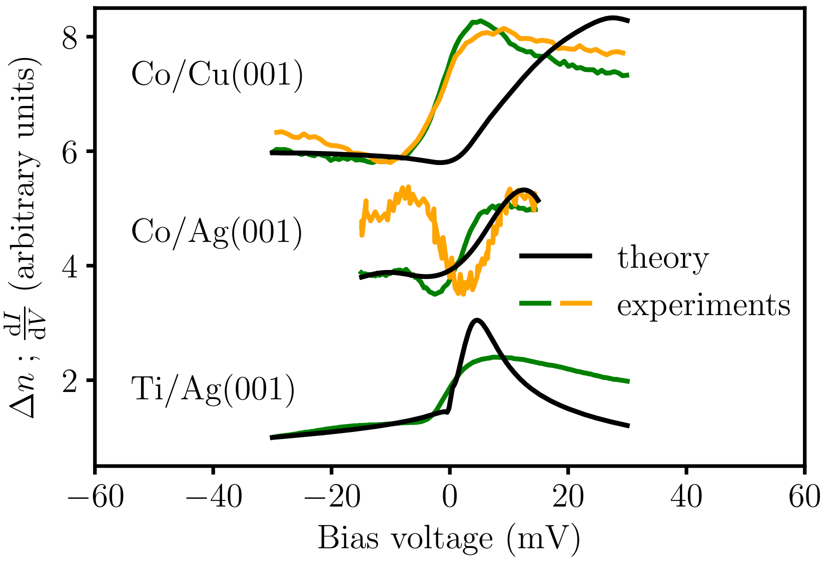

Supplementary Figure 1: Zero-bias anomalies calculated and measured on Co adatoms on Cu and Ag(001) surfaces as well as Ti adatom on Ag(001) surface. For Co/Cu(001), the agreement with the experimental data [1, 2] is not as perfect as it is on the (111) surfaces. We recover, however, the observed step-like behavior. A reason could be the underestimation of the magnetic anisotropy energy of Co on Cu(001), which can shift slightly the spectrum around. For Co/Ag(001), the agreement is rather good when compared to the data of Ternes et al. [3]. It is interesting to notice, however, that the measurements of Wahl et al. [4] lead to different spectra. The disagreement between the two experimental data can be surprising at first sight. We conjecture that this can be induced by the presence of hydrogen or by the difference in the probing tip, which can change the shape of the features. This strongly motivates further experimental investigations. The case of of Ti/Ag(001) measured by measured by Nagaoka et al. [5] is rather reasonably described by our theory. Our zero-bias anomaly is sharper than the experimental one. Overall, the agreement between theory and experiment is rather good on the (001) surfaces, which indicates that spin-excitations, as discussed in the main text, is a plausible origin of the low-energy features on the (001) surfaces of Cu and Ag, similarly to the (111) surfaces. Note that the position of the theoretically obtained features hinges on the ability to evaluate the magnetic anisotropy energy of the adatoms, which is not always trivial. The experimental data adapted with permission from Refs. 1–5. Copyright (2002), (2004) and (2007) by the American Physical Society and (2008) by IOP Publishing.

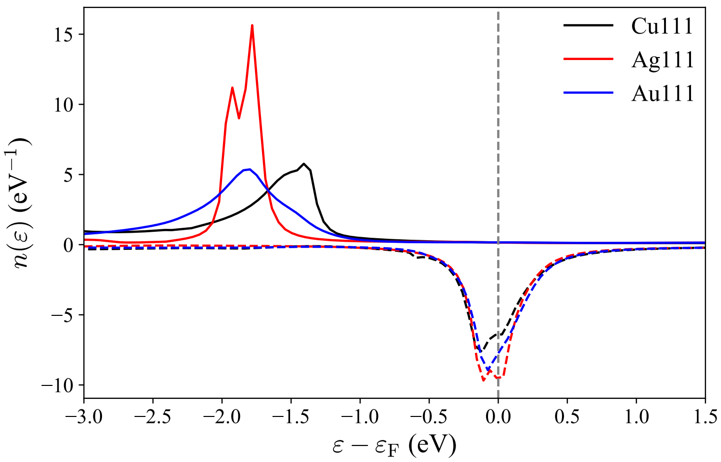

Supplementary Figure 2: Total spin-polarized local density of states of a Co adatom on Cu, Ag and Au(111) surfaces. The minority-spin state (lower panel) for Co on Ag(111) is sharper due to the weaker strength of the hybridization of the electronic states of the adatom and the substrate, when compared to Au or Cu(111).

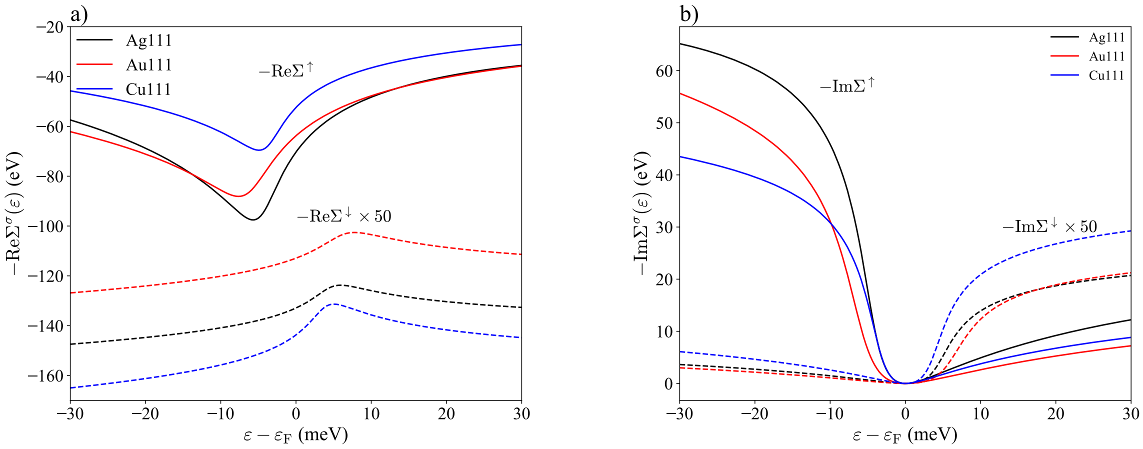

Supplementary Figure 3: Comparison of the spin-resolved imaginary and real parts of the trace of the Co adatoms self-energies on the three surfaces Cu, Ag, Au(111). Similarly to Au(111) surface (shown also in the main Figure 2b), the steps characterizing the imaginary part of the self-energies are asymmetric. The one generated at negative bias voltage corresponding to the majority-spin channel is the largest on Ag. This is induced by the large minority-spin local density of states (see Supplemental Figure 2), which defines the height of the step.

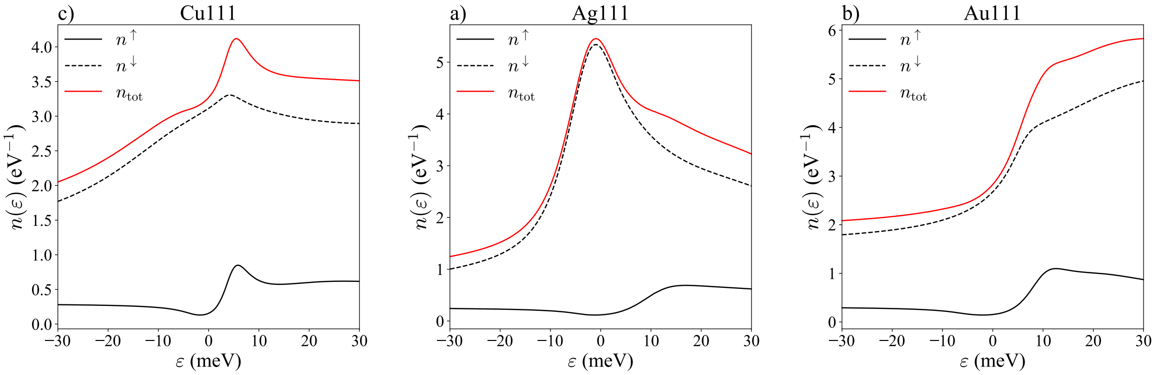

Supplementary Figure 4: Comparison of the renormalized local density of states of Co adatoms on the three surfaces Cu, Ag, Au(111). Black full (dashed) lines correspond to the majority-spin (minority-spin states), while the full red lines depict the total density of states. The theoretical inelastic spectra are calculated in the vacuum as a result of the decay of adatoms electronic states. Therefore the shape of the signature of the spin-excitations and of the spinaron do not have to be the same in the adatom and in the vacuum.

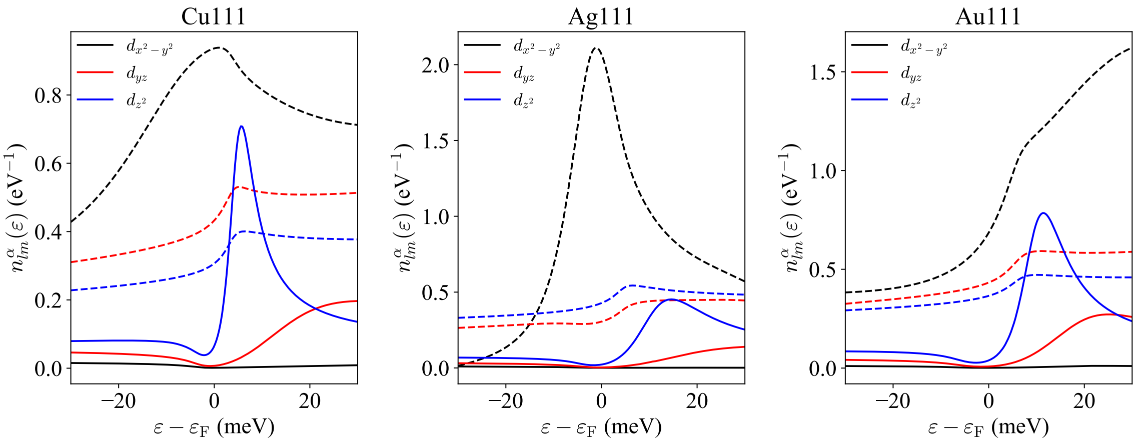

Supplementary Figure 5: Orbital-resolved renormalized local density of states of Co adatoms on the three surfaces Cu, Ag, Au(111). Full (dashed) lines correspond to the majority-spin (minority-spin) channel. The spinaron emerges from the majority-spin d_{z^2} orbital (full blue line).

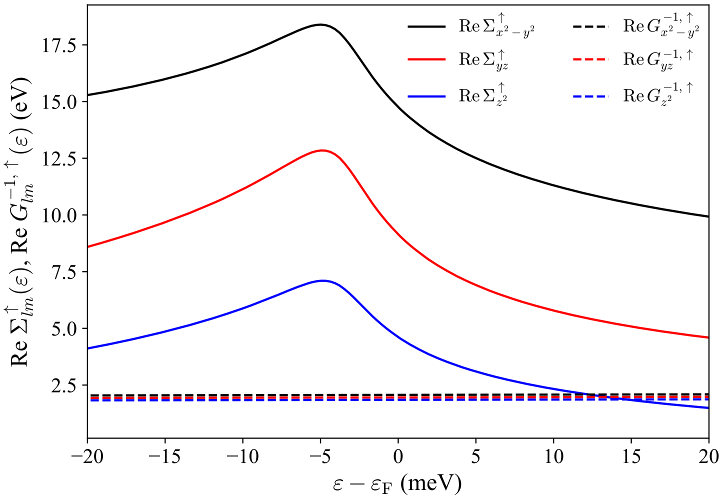

Supplementary Figure 6: Orbital-resolved real part of inverse Green function and self-energy of the majority spin channel of Co adatoms on Cu(111) surface. The intersection occurring in the d_{z^2}-orbital leads to a vanishing denominator of the Eq 1 of the main text and, therefore, to the spinaron. The contribution of $\mathrm{Im}[\underline{G}(\varepsilon)]\mathrm{Im}[\underline{\Sigma}(\varepsilon)]$ (and other interference effects) shifts the spinaron to lower energies.

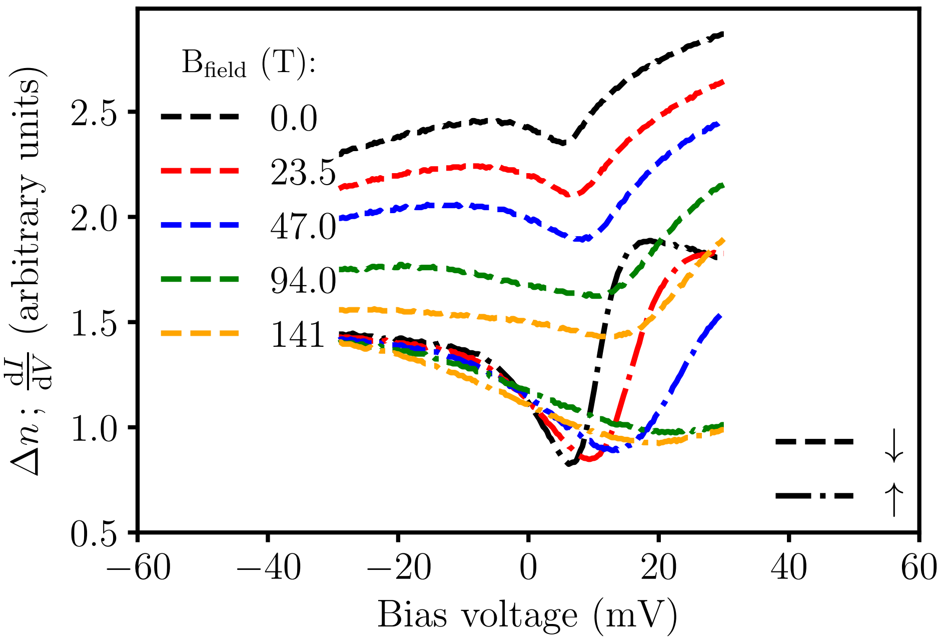

Supplementary Figure 7: Theoretical scanning tunneling spectroscopy spectra probing the differential conductance of a Co adatom on Au(111). After applying a magnetic field, the spin-resolved spectra of the zero-bias signals (shown in Fig. 3C of the main text) experience various changes. The signature of the spin-excitation in the minority-spin channel moves to larger energies, as expected. The spinaron, living in the majority-spin channel, also shifts to higher energies. This occurs as a result of the increase of the spin-excitation gap upon application of the magnetic field. The intrinsic spin-excitation expected at negative bias voltage should shift to lower energies as well. However, it is difficult to track its low signal, that is caused by the large imaginary part of the corresponding self-energy, which considerably lowers the lifetime of the majority-spin electrons. In other words, the expected feature is much broader than the one occurring in the minority-spin channel. Overall, we notice that all features experience a decrease in their lifetimes since more electron-hole excitations are allowed when increasing the excitation energy. Summing up both spin-channels, as done in Fig. 3C, leads to two steps with a dip in-between. The dip shifts to higher energies, while the gap increases in magnitude after application of the magnetic field.

Supplementary Note 1 Description of the formalism used to describe the self-energy

Supplementary Note 1.1 General form of the self-energy.

Utilizing many-body perturbation theory, the self-energy describing the interaction of electrons and spin-excitations can be written in the following form:

$$\Sigma^{\sigma\sigma'}(\mathbf{r},\mathbf{r}',\varepsilon) = -\frac{U(\mathbf{r})\,U(\mathbf{r}')}{\pi}\sum_{ss'}\left\{\int_0^{\infty} d\omega\,\mathrm{Im}\left[G^{ss'}(\mathbf{r},\mathbf{r}',\varepsilon+\omega)\,\chi^{\sigma s,s'\sigma'}(\mathbf{r},\mathbf{r}',\omega)\right]\right.$$
$$\left. - \int_0^{\varepsilon_{\mathrm{F}}-\varepsilon} d\omega\,\mathrm{Im}\left[G^{ss'}(\mathbf{r},\mathbf{r}',\varepsilon+\omega)\right]\left[\chi^{\sigma s,s'\sigma'}(\mathbf{r}',\mathbf{r},\omega)\right]^*\right\} \quad , \tag{S.1}$$

which is a generalized form [6] of the self-energy described in Ref. 7, where spin-orbit interaction is included, leading to possible contributions (in spin-space) of off-diagonal elements of the Green functions and of the tensor of the dynamical spin-susceptibility. $\chi^{\sigma s,s'\sigma'}(\omega)$ is the Fourier transform of the spin-susceptibility $\chi^{\sigma s,s'\sigma'}(t) = -\mathrm{i}\,\Theta(t)\langle[\hat{S}_{\sigma s}(t),\hat{S}_{s'\sigma'}]\rangle$, and $\sigma,\sigma',s,s' = \{\uparrow,\downarrow\}$. The latter can be identified as the susceptibility obtained from time-dependent density functional theory (TD-DFT) while $U(\vec{r})$ represents the exchange-correlation kernel, evaluated in the adiabatic local spin-density approximation (ALSDA) [7]. The self-energy consists then on two kinds of elements: spin-diagonal and off-diagonal contributions.

Supplementary Note 1.2 Diagonal spin-components of the self-energy and approximations.

The diagonal spin-terms of the self-energy are $\Sigma^{\uparrow\uparrow}$ and $\Sigma^{\downarrow\downarrow}$. The former is schematically given by

$$\begin{aligned}\Sigma^{\uparrow\uparrow}(\varepsilon) \propto &\sum_{ss'}\chi^{\uparrow s,s'\uparrow}(\omega)\,G^{ss'}(\omega+\varepsilon)\\ \propto&\chi^{\uparrow\uparrow,\uparrow\uparrow}(\omega)\,G^{\uparrow\uparrow}(\omega+\varepsilon)+\chi^{\uparrow\uparrow,\downarrow\uparrow}(\omega)\,G^{\uparrow\downarrow}(\omega+\varepsilon)\\ &+\chi^{\uparrow\downarrow,\uparrow\uparrow}(\omega)\,G^{\downarrow\uparrow}(\omega+\varepsilon)+\chi^{\uparrow\downarrow,\downarrow\uparrow}(\omega)\,G^{\downarrow\downarrow}(\omega+\varepsilon)\end{aligned} \tag{S.2}$$

The bias voltages that are addressed experimentally to probe spin-excitations of adatoms via inelastic scanning tunneling spectroscopy (ISTS) are located in the meV range, where the transverse spin-excitations are located. These modes are encrypted in the transverse (spin-flip) block of the susceptibility. For the systems we investigate, the off-diagonal elements of the susceptibility tensor, i.e. the transverse-longitudinal block, is found negligible, i.e. $\chi^{\uparrow\uparrow,\downarrow\uparrow}(\omega) \simeq \chi^{\uparrow\downarrow,\uparrow\uparrow}(\omega) \simeq 0$. The longitudinal component $\chi^{\uparrow\uparrow,\uparrow\uparrow}(\omega)$ encodes excitations located at higher frequencies (eV range), so its contribution is neglected for the investigated frequencies. Therefore, $\Sigma^{\uparrow\uparrow}(\varepsilon)$ is simply given by

$$\Sigma^{\uparrow\uparrow}(\varepsilon) \propto \chi^{+,-}(\omega)\,G^{\downarrow\downarrow}(\omega+\varepsilon) \quad , \tag{S.3}$$

and similarly

$$\Sigma^{\downarrow\downarrow}(\varepsilon) \propto \chi^{-,+}(\omega)\,G^{\uparrow\uparrow}(\omega+\varepsilon) \quad , \tag{S.4}$$

where the usual short notation $\chi^{+,-}(\omega) = \chi^{\uparrow\downarrow,\downarrow\uparrow}(\omega)$ and $\chi^{-,+}(\omega) = \chi^{\downarrow\uparrow,\uparrow\downarrow}(\omega)$ has been employed. We note that our extended approach includes the intrinsic gap opening in the spin-excitation spectrum due to the breaking of rotational symmetry induced by spin-orbit interaction.

Supplementary Note 1.3 Off-diagonal spin-components of the self-energy and approximations.

The spin off-diagonal contribution to the self-energy reads

$$\Sigma^{\downarrow\uparrow}(\varepsilon) \propto \chi^{\downarrow\uparrow,\uparrow\uparrow}(\omega)\, G^{\uparrow\uparrow}(\omega+\varepsilon) + \chi^{\downarrow\uparrow,\downarrow\uparrow}(\omega)\, G^{\uparrow\downarrow}(\omega+\varepsilon) + \chi^{\downarrow\downarrow,\uparrow\uparrow}(\omega)\, G^{\downarrow\uparrow}(\omega+\varepsilon) + \chi^{\downarrow\downarrow,\downarrow\uparrow}(\omega)\, G^{\downarrow\downarrow}(\omega+\varepsilon)\ . \tag{S.5}$$

As aforementioned, the off-diagonal longitudinal-transverse block and the longitudinal contributions of the susceptibility tensor have negligible contributions for the investigated adatoms. The remaining term involving the component $\chi^{-,-}(\omega) = \chi^{\downarrow\uparrow,\downarrow\uparrow}(\omega)$ vanishes in systems with C_{3v} symmetry or higher, as the ones investigated in our work [8]. Therefore, the off-diagonal components of the self-energy $\Sigma^{\downarrow\uparrow}(\varepsilon)$ and $\Sigma^{\uparrow\downarrow}(\varepsilon)$ can be safely neglected for the specific cases investigated in the main manuscript.

Supplementary Note 1.4 Forms of the self-energies shown in the manuscript.

The self-energy used in practical calculations has a spin-diagonal form. For an applied bias voltage V, the real part of the spin-diagonal components of the self-energy read

$$\begin{aligned}\mathrm{Re}\,\Sigma^{\sigma\sigma}(\mathbf{r},\mathbf{r}',\varepsilon_\mathrm{F}+V) = -\frac{U(\mathbf{r})\,U(\mathbf{r}')}{\pi}\Bigg\{ &\int_0^{\infty} d\omega\, \mathrm{Im}\left[G^{\bar\sigma\bar\sigma}(\mathbf{r},\mathbf{r}',\varepsilon_\mathrm{F}+V+\omega)\,\chi^{\sigma\bar\sigma,\bar\sigma\sigma}(\mathbf{r},\mathbf{r}',\omega)\right] \\ &- \int_0^{-V} d\omega\, \mathrm{Im}\left[G^{\bar\sigma\bar\sigma}(\mathbf{r},\mathbf{r}',\varepsilon_\mathrm{F}+V+\omega)\right] \mathrm{Re}\left[\chi^{\sigma\bar\sigma,\bar\sigma\sigma}(\mathbf{r}',\mathbf{r},\omega)\right]\Bigg\}\quad .\end{aligned} \tag{S.6}$$

Note that the first term in Eq. S.6 involves a frequency integration up to infinity. In practice, the evaluation of the integral is done in two steps: First, the magnetic susceptibility is computed up to a cut-off frequency $\omega_\mathrm{cut} = 100\,\mathrm{meV}$. The value of the cut-off is chosen after systematic convergence calculations. Second, for the remaining range, we make use of the high-frequency expansion of the spin-flip susceptibility [9]

$$\begin{aligned}\lim_{\omega\to+\infty} \mathrm{Re}\,\chi_{+-}(\omega) &\approx \frac{C^\mathrm{Re}}{\omega} + \ldots \quad , \\ \lim_{\omega\to+\infty} \mathrm{Im}\,\chi_{+-}(\omega) &\approx \left(\frac{C^\mathrm{Im}}{\omega^2}\right) + \ldots \quad ,\end{aligned} \tag{S.7}$$

where $C^\mathrm{Re} = \mathrm{Re}\,\chi_{+-}(\omega_\mathrm{cut})\,\omega_\mathrm{cut}$ and $C^\mathrm{Im} = \mathrm{Im}\,\chi_{+-}(\omega_\mathrm{cut})\,\omega_\mathrm{cut}^2$.

The imaginary part of the self-energy is simply given by

$$\mathrm{Im}\,\Sigma^{\sigma\sigma}(\mathbf{r},\mathbf{r}',\varepsilon_\mathrm{F}+V) = -\frac{U(\mathbf{r})\,U(\mathbf{r}')}{\pi}\int_0^{-V} d\omega\, \mathrm{Im}\left[G^{\bar\sigma\bar\sigma}(\mathbf{r},\mathbf{r}',\varepsilon_\mathrm{F}+V+\omega)\right] \mathrm{Im}\left[\chi^{\sigma\bar\sigma,\bar\sigma\sigma}(\mathbf{r}',\mathbf{r},\omega)\right]. \tag{S.8}$$

For the bias voltages applied experimentally ($\sim$ meV), the imaginary part of the Green function of the investigated adatoms depends weakly on the energy. As a result, the imaginary part of the self-energy presents a step as function of the bias voltage when integrating over the peak position of the susceptibility. Thus the position of the step is the hallmark of the excitation energy.

Supplementary Note 1.5 Projection basis for the Green function and susceptibility

The aforementioned self-energy is given as a convolution of the single particle Green function and the spin-spin susceptibility. In practice, these quantities are evaluated from the Korringa-Kohn-Rostoker (KKR) Green function approach using the atomic sphere approximation (ASA) [10–12]. Within this method, the space is partitioned into cells centred around atomic sites:

$$G_{i,j}^{\sigma\sigma'}(\mathbf{r},\mathbf{r}',\varepsilon) = \sum_{L,L'} Y_L(\hat{\mathbf{r}})\, G_{iL,jL'}^{\sigma\sigma'}(r,r',\varepsilon)\, Y_{L'}(\hat{\mathbf{r}}') \quad , \tag{S.9}$$

the distance r (r') is measured from the atomic site i (j). The angular dependence is expanded in real spherical harmonics $Y_L(\hat{\mathbf{r}})$, with $L = \{l, m\}$ being the azimuthal and magnetic quantum numbers, respectively. Furthermore, to access complex quantities such as response functions a projection basis is introduced in practice [12, 13]. The latter is built from the spin-independent regular solutions $R_{il}(r, \varepsilon)$:

$$\phi_{ilb}(r) = \frac{R_{il}(r, \varepsilon_\mathrm{b})}{\sqrt{\int_0^R dr\, r^2\, R_{il}(r, \varepsilon_\mathrm{b})^2}} \quad . \tag{S.10}$$

ε_b represents a set of energies chosen in the the valence states energy range. The radial part of the Green function is the expressed in the the projection basis as

$$G^{\sigma\sigma'}_{iL,jL'}(r, r', \varepsilon) = \sum_{bb'} \phi_{ilb}(r)\, G^{\sigma\sigma'}_{iLb,jL'b'}(\varepsilon)\, \phi_{jl'b'}(r') \quad . \tag{S.11}$$

b and b' define the basis indices.

The convolution of two single-particle Green functions gives rise to the Kohn-Sham magnetic susceptibility [12, 13]. Once more, to reduce the computational costs, we introduce a mixed product basis $\Phi_{iLb}(x)$ by contracting the product of the Green function basis [13, 14]:

$$\sum_b C^{iLb}_{L_1 b_1 L_2 b_2} \Phi_{iLb}(r) = C^L_{L_1 L_2} \phi_{il_1 b_1}(r)\, \phi_{il_2 b_2}(r) \quad . \tag{S.12}$$

$C^L_{L_1 L_2}$ and $C^{iLb}_{L_1 b_1 L_2 b_2}$ represent gaunt and generalized Gaunt coefficients, respectively. In this new basis, the response function reads:

$$\chi^{\alpha\beta}_{ij}(\mathbf{r}, \mathbf{r}', \omega) = \sum_{Lb, L'b'} \Phi_{iLb}(r)\, Y_L(\hat{\mathbf{r}})\, \chi^{\alpha\beta}_{iLb,jL'b'}(\omega)\, \Phi_{jL'b'}(r')\, Y_L(\hat{\mathbf{r}}') \quad . \tag{S.13}$$

Finally, we provide the basis representation of the self-energy, which is schematically proportional to: $\Sigma_{ij}(\mathbf{r}, \mathbf{r}', \varepsilon) \propto U_i(\mathbf{r})\, G_{ij}(\mathbf{r}, \mathbf{r}', \varepsilon)\, \chi_{ij}(\mathbf{r}, \mathbf{r}', \varepsilon')\, U_j(\mathbf{r}')$. Following the expansion chosen for the Green function as defined in Eq. S.10, the self-energy reads:

$$\Sigma_{i,j}(\mathbf{r}, \mathbf{r}', \varepsilon) = \sum_{Lb, L'b'} Y_L(\hat{\mathbf{r}})\, \phi_{ilb}(r)\, \Sigma_{iLb,jL'b'}(\varepsilon)\, \phi_{jl'b'}(r')\, Y_{L'}(\hat{\mathbf{r}}') \quad , \tag{S.14}$$

with the self-energy components given by

$$\Sigma_{iL_1b_1,jL_2b_2}(\varepsilon) = \sum_{Lb, L'b'} \sum_{\substack{L_3b_3, L_4b_4 \\ L_5b_5, L_6b_6}} C^{iL_3b_3}_{L_1b_1Lb} C^{jL_4b_4}_{L_2b_2L'b'} U_{i,L_3b_3,L_5b_5}\, G_{iLb,jL'b'}(\varepsilon)\, \chi_{iL_5b_5,jL_6b_6}(\varepsilon')\, U_{j,L_6b_6,L_4b_4} \tag{S.15}$$

Finally, assuming that the kernel is diagonal in the basis, and using the spherical average of the magnetic susceptibility and of the exchange-correlation kernel [13], the previous equation reduces to

$$\Sigma_{iL_1b_i,jL_2b_j}(\varepsilon) = \sum_{bb'b_3b_4} C^{i0b_3}_{L_1b_iL_1b1}\, C^{j0b_4}_{L_2b_jL_2b_2}\, U_{i,0b_3}\, G_{iL_1b_1,jL_2b_2}(\varepsilon)\, \chi_{i0b_3,j0b_4}(\varepsilon')\, U_{j,0b_4} \quad . \tag{S.16}$$

Supplementary Note 2 Simplified scheme with an auxiliary magnetic field instead of magnetic anisotropy

We note that the case of Co adatom on Cu(111) was addressed in the preliminary work reported in Ref. 7, where the self-energies of the adatoms were computed in a very simplified approach compared to the current

work. The results are, however, in line with those obtained with the more accurate formalism described in our manuscript. In Ref. 7, a rudimentary projection basis (based on regular scattering solutions that are computed at the Fermi energy) was utilized, which is not that precise to describe various spin-dynamics properties. More importantly, it was lacking the impact of the spin-orbit coupling. The simplest implication of this interaction is to open a gap of a few meV in the susceptibility, which has to be precisely described. Instead of spin-orbit coupling, a magnetic field was utilized to effectively open a gap in the spin-excitations spectra. The former has profoundly different implications on the dynamical susceptibilities compared to those induced by the latter. The main ingredient is the definition of the correct ground state of the magnetic orientation with respect to the lattice, which is not known when magnetic fields are used. Additionally, the magnitude of the field is arbitrary and has a different physical origin. These aspects induce drastic modifications in the properties of the susceptibilities — and therefore in the self-energy and the resulting renormalized electronic structure: the shape and weight of the spin-flip relativistic responses, χ^{+-} and χ^{-+}, at positive and negative frequencies (both imaginary and real parts) change dramatically if the moment is lying in an easy-plane or along an easy axis, depending also if it is in the ground state or in a metastable state. Consequently, it is impossible to make any reasonable claim and systematic interpretation of the experimental data of Co on the three noble metallic substrates (Cu, Ag, Au) from such a poor's man approach. These issues were solved by our current approach.

Supplementary References

[1] Knorr, N., Schneider, M. A., Diekhöner, L., Wahl, P. & Kern, K. Kondo Effect of Single Co Adatoms on Cu Surfaces. *Phys. Rev. Lett.* **88**, 096804 (2002).

[2] Wahl, P. *et al.* Exchange Interaction between Single Magnetic Adatoms. *Phys. Rev. Lett.* **98**, 056601 (2007).

[3] Ternes, M., Heinrich, A. J. & Schneider, W.-D. Spectroscopic manifestations of the Kondo effect on single adatoms. *Journal of Physics: Condensed Matter* **21**, 053001 (2008).

[4] Wahl, P. *et al.* Kondo Temperature of Magnetic Impurities at Surfaces. *Phys. Rev. Lett.* **93**, 176603 (2004).

[5] Nagaoka, K., Jamneala, T., Grobis, M. & Crommie, M. F. Temperature Dependence of a Single Kondo Impurity. *Phys. Rev. Lett.* **88**, 077205 (2002).

[6] Fransson, J. Inelastic-impurity-scattering-induced spin texture and topological transitions in surface electron waves. *Phys. Rev. B* **92**, 125405 (2015).

[7] Schweflinghaus, B., dos Santos Dias, M., Costa, A. T. & Lounis, S. Renormalization of electron self-energies via their interaction with spin excitations: A first-principles investigation. *Phys. Rev. B* **89**, 235439 (2014).

[8] Guimarães, F. S. M., dos Santos Dias, M., Schweflinghaus, B. & Lounis, S. Engineering elliptical spin-excitations by complex anisotropy fields in Fe adatoms and dimers on Cu(111). *Phys. Rev. B* **96**, 144401 (2017).

[9] Giuliani, G. & Vignale, G. *Quantum theory of the electron liquid*, vol. 9780521821124 (Cambridge University Press, 2005), 1 edn.

[10] Papanikolaou, N., Zeller, R. & Dederichs, P. H. Conceptual improvements of the KKR method. *Journal of Physics: Condensed Matter* **14**, 2799 (2002).

[11] Bauer, D. S. G. Development of a relativistic full-potential first-principles multiple scattering Green function method applied to complex magnetic textures of nanostructures at surfaces. *Forschungszentrum Jülich* (2014).

[12] Lounis, S., Costa, A. T., Muniz, R. B. & Mills, D. L. Theory of local dynamical magnetic susceptibilities from the Korringa-Kohn-Rostoker Green function method. *Phys. Rev. B* **83**, 035109 (2011).

[13] dos Santos Dias, M., Schweflinghaus, B., Blügel, S. & Lounis, S. Relativistic dynamical spin excitations of magnetic adatoms. *Phys. Rev. B* **91**, 075405 (2015).

[14] Aryasetiawan, F. & Gunnarsson, O. Product-basis method for calculating dielectric matrices. *Phys. Rev. B* **49**, 16214–16222 (1994).
